# Accessing Mefenamic Acid Form II through High-Pressure Recrystallisation

**DOI:** 10.3390/pharmaceutics9020016

**Published:** 2017-05-16

**Authors:** Nasir Abbas, Iain D. H. Oswald, Colin R. Pulham

**Affiliations:** 1 College of Pharmacy, University of the Punjab, Lahore 54000, Pakistan; 2 Strathclyde Institute of Pharmacy and Biomedical Sciences, University of Strathclyde, 161 Cathedral Street, Glasgow G4 0RE, UK; iain.oswald@strath.ac.uk; 3 School of Chemistry, University of Edinburgh, David Brewster Road, Edinburgh EH9 3FJ, UK; c.r.pulham@ed.ac.uk

**Keywords:** mefenamic acid, diamond anvil cell, high-pressure, polymorphism, high-pressure crystallisation, non-steroidal anti-inflammatory drugs

## Abstract

High-pressure crystallisation has been successfully used as an alternative technique to prepare Form II of a non-steroidal anti-inflammatory drug, mefenamic acid (MA). A single crystal of Form II, denoted as high-pressure Form II, was grown at 0.3 GPa from an ethanolic solution by using a diamond anvil cell. A comparison of the crystal structures shows that the efficient packing of molecules in Form II was enabled by the structural flexibility of MA molecules. Compression studies performed on a single crystal of Form I resulted in a 14% decrease of unit cell volume up to 2.5 GPa. No phase transition was observed up to this pressure. A reconstructive phase transition is required to induce conformational changes in the structure, which was confirmed by the results of crystallisation at high pressure.

## 1. Introduction

The phenomenon of polymorphism in a solid state is the ability of a substance to crystallise with more than one crystal form; these forms are called polymorphs. The polymorphism of organic compounds is of fundamental interest to experimental and theoretical chemists, and is also of crucial importance to industry for a wide range of materials that include pharmaceuticals, pigments, explosives (energetic materials), food products and proteins [1,2]. In pharmaceuticals, polymorphism of the active pharmaceutical ingredient (API) is of great importance. Differences in the three-dimensional arrangements and/or conformations of molecules can have profound effect on physical and chemical properties such as the melting point, sublimation temperature, heat capacity, solubility, dissolution and bio-availability of the API [2]. Many processing properties are also dependent on the polymorphic form of the API [1]. Polymorphic inter-conversion during the manufacturing process (such as compression, milling and drying) and the storage of drug compounds is also of crucial importance, where changes in conditions, e.g. temperature, relative humidity and pressure, may result in the formation of new forms. Patent rights also become an issue, as polymorphs of the same compound can be patented separately. The cases of Ritonavir (an HIV drug by Abbot Laboratories, North Chicago, IL, USA) and ranitidine hydrochloride (Zantac, the largest selling anti-ulcer drug from GlaxoSmithKline, Brentford, Middlesex, UK), provide examples of the importance of polymorphism in processing and patent litigation, where the emergence of new polymorphs resulted in huge financial loss to the companies [3,4]. For all of the above reasons, pharmaceutical companies deploy great effort and resources for the identification of all possible polymorphs of a compound in the early stage of its development.

In recent years, considerable effort has been put into developing high-throughput, automated approaches to polymorph screening where key crystallisation variables such as solvent and anti-solvent selection, evaporation and cooling rate, followed by characterisation by spectroscopic, diffraction and thermal techniques, are employed [5]. The main aim is to develop methods that are capable of producing and identifying large numbers of samples in a relatively short period of time. Even after these exhaustive tests, a new polymorph may remain undetected for many years, or a sample of a new polymorph may be obtained once but never again, the phenomenon of so-called “disappearing polymorphs” [6,7]. Polymorphism remains one of the major challenges for both the industry and academic sectors. The development of complementary methods with better control on the crystallisation conditions, and more importantly the understanding of factors that result in different packing arrangements of organic molecules in the solid state would therefore be highly desirable [8].

Hence, there is a need to explore alternative crystallisation conditions, e.g., use of pressure, as an additional variable in conjunction with conventional strategies. Polymorph screening is normally performed under ambient conditions of pressure (0.0001 GPa). Pressure can change the relative thermodynamic stability of polymorphs and also can have effect on the conformation of molecules as they pack within a crystal structure. It can act as an additional variable for the identification of new polymorphs. High pressure has already been extensively applied in polymorphic studies of many simple organic compounds such as amines, ketones, carboxylic acids, alcohols [9,10] and amino acids [11], where direct compression has successfully been used to generate new polymorphs. However, on occasion compression is not enough for the compounds where the kinetic barrier of molecular rearrangement is high and cannot be achieved by simple compression. To overcome this problem, a technique has been developed which involves the recrystallisation of a compound from a solution under elevated pressure (i.e. pressure-induced crystallisation). This has successfully been used to generate new polymorphs of simple compounds, e.g. new polymorphs of parabanic acid [8], urea [12], acetamide, and paracetamol [13].

Mefenamic acid (MA) (2-[(2,3-dimethyphenyl)-amino] benzoic acid), is a well-known fenamate derivative showing potent analgesic effect [14], and is marketed as Ponstel^®^. It belongs to the group of medicines called non-steroidal anti-inflammatory drugs (NSAIDs), which are among the most frequently used medicinal drugs. MA is known to exist in three polymorphic forms, denoted as Forms I, II and III [15]. These polymorphs show different solubility and stability behaviours [16,17]. Form I is the most stable form under ambient conditions, Form II is the stable form above 160 °C and Form III is the least stable form at ambient conditions and converts back to Form I immediately [15]. The crystal structures of Form I, II and III were reported in 1976, 2008 and 2012, respectively [15,18,19]. NMR spectroscopy and theoretical studies have shown that Form I has some conformational similarities with related fenamate compounds e.g., flufenamic acid, niflumic acid and maclofenamic acid [20]. All polymorph screening of MA has been performed at ambient pressure [21]. Furthermore, an MA molecule shows structural flexibility that can instigate more conformational polymorphs using high-pressure conditions. All of the above characteristics make MA an ideal molecule for high-pressure studies. Therefore, the aim of current study is to explore the effect of pressure on the polymorphic behavior of MA. At the first stage, the effect of hydrostatic pressure on the polymorphic transition of a single crystal of the most stable polymorph, i.e. Form I, is studied. Subsequently, the high-pressure crystallisation technique is employed to investigate the crystallisation and packing of MA molecules in the crystal structure at elevated pressure conditions.

## 2. Materials and Methods

Mefenamic acid was obtained from the Sigma Aldrich, Dorset, UK. Single crystals of Form I were obtained by the slow cooling of a hot saturated solution (ca. 1 M) in THF. A small, colourless, block-shaped crystal was taken directly from the crystallised sample and was confirmed as being Form I by indexing the diffraction data from single crystal X-ray diffraction. Crystals from the same solution were used in all of the single crystal compression experiments. Analytical grade solvents were used for the recrystallisation experiments.

High-pressure experiments were carried out using a Merrill-Bassett diamond anvil cell (DAC) (Almex easyLab, Reading, UK) (half-opening angle 40°) [22], equipped with 600-μm culet diamonds, a Beryllium backing disc and a pre-indented tungsten gasket with a thickness of 250 µm and a diameter hole of 300 µm. For high-pressure crystallisation experiments, diamond anvil cells using tungsten carbide backing discs [23] were used.

### 2.1. Compression Studies

For compression studies, a single crystal of Form I of suitable size was loaded into a DAC along with a small piece of ruby in order to allow for the determination of the pressure by laser fluorescence method [24] using a 632.8 nm excitation line from a He-Ne laser. The fluorescence was detected by a Jobin-Yvon LabRam 300 (Horiba Scientific, Stanmore Middlesex, UK). A methanol-ethanol (4:1) mixture was used as the pressure-transmitting medium. It is one of the most commonly used pressure-transmitting media for single crystal diffraction studies, which is believed to remain at least quasi hydrostatic to its glass transition at 10.4 GPa [25].

### 2.2. Crystallisation at High Pressure

For high-pressure crystallisation, a ca. 1 M solution of mefenamic acid was prepared in ethanol. Ethanol was selected as a solvent on the basis of MA solubility. The solution was then loaded along with a few crystallites of Form I at 293(2) K into a DAC. The pressure was increased to a minimum (~0.02 GPa) to form a sealed system and the cell was then gently heated to dissolve all of the solid material. Pressure was then applied by tightening the screws of the DAC to induce precipitation whilst still hot. Crystallisation was achieved at ca. 0.6 GPa. The pressure was then reduced to 0.3 GPa in order to facilitate the growth of a single crystal. The temperature was then cycled near 353 K in order to dissolve all but one of the crystallites. On slow cooling to 298 K, a single crystal grew inside the gasket chamber.

### 2.3. Single Crystal X-ray Diffraction

Diffraction data were collected on a Bruker APEX II CCD diffractometer (Bruker, Coventry, UK) at 293(2) K using Mo-Kα radiation (λ = 0.71073). Data indexing was performed using CELL_NOW [26]. Data processing was performed according to the procedure described by Dawson et al. (2004) [27]. Integration of datasets and global cell refinement were carried out using the program SAINT [28], in which ′dynamic masks′ were used in order to prevent the integration of areas of the detector shaded by the body of the DAC. An analytical correction for the absorption by the DAC component was then applied using SHADE [29] and an absorption correction for the crystal was applied using SADABS [30]. Known coordinates were taken from the literature [18]. Refinement was then performed using CRYSTALS [31]. All non-hydrogen atoms were refined isotropically and hydrogen atoms were placed in calculated positions. A completeness of only ca. 40% was obtained for this dataset, and this led to a rather high *R*-factor of 9.5%. Nevertheless, this was sufficient to identify the main structural feature of Form II and make a comparison between the polymorphs. Full structural refinements details and crystallographic data in CIF format is attached as Supplementary Materials.

### 2.4. Infrared Spectroscopy

Infrared (IR) spectra of samples were recorded using FT-IR spectrophotometer (Alpha-P Bruker, Ettlingen, Germany) equipped with an ATR (Attenuated Total Reflection) unit across the range of 4000–400 cm^−1^ .

## 3. Results

### 3.1. Direct Compression of a Single Crystal of Form I

The first dataset was collected at ambient pressure. Indexing of the reflections confirmed the known triclinic Form I of MA (the lattice parameters reported in Table 1 were obtained as a standard setting i.e., smallest dimension in *a*, then largest in *c*). Diffraction data were then collected in incremental steps of pressure up to 2.5 GPa. The lattice parameters obtained at different pressures are shown in Table 1. On raising the pressure to 3.0 GPa, the crystal disintegrated.

**Figure 1:**
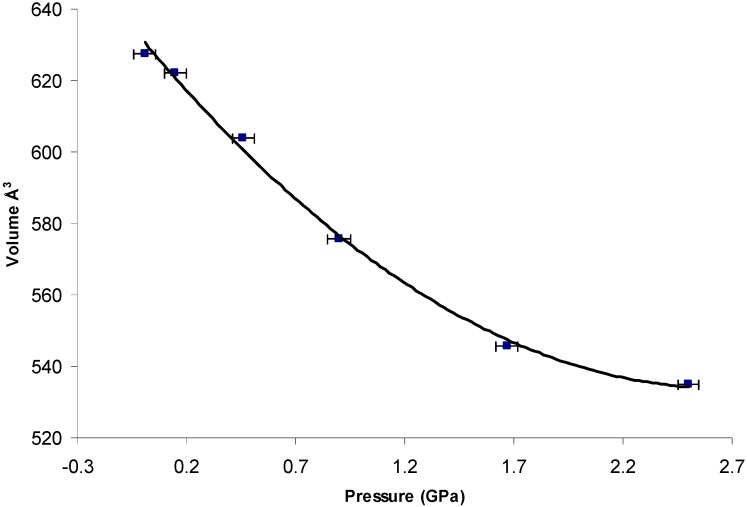
Variation of the unit cell volume of Form I with pressure.

All datasets up to 2.50 GPa were processed according to the procedure described above. As a result of the limitations caused by shading from the steel body of the diamond anvil cell, high-pressure datasets are frequently incomplete compared with datasets recorded at ambient pressure. These factors combined to make structural refinement particularly challenging, and in this case resulted in poor structural refinement with high *R*-factors. Therefore, only lattice parameters are reported here. Figure 1 shows the variation of the unit cell volume with increasing pressure. There was a ca. 14% decrease in the unit cell volume over the studied pressure range. Within the limits of experimental errors, the volume decreased smoothly over this pressure range, indicating that there is no phase transition associated with the direct compression of the single crystal.

The observation that at 3.0 GPa the crystal disintegrated to give a polycrystalline powder suggested a reconstructive phase transition at this pressure. This observation prompted a study by recrystallisation from solution at high pressure.

#### 3.1.1. Crystallisation from Solution at High Pressure

Crystallisation from ethanolic solution was achieved at 0.6 GPa. Pressure was then reduce to 0.3 GPa to facilitate the growth of a single crystal. The crystal structure obtained from the single crystal grown at 0.3 GPa resulted in a triclinic unit cell (Table 2), which turns out to be Form II (the lattice parameters are comparable with Form II); this is denoted as high-pressure Form II. Crystallographic data obtained from the high-pressure Form II was compared with already published forms I, II and III (Table 2; Table S1). All three polymorphs (I−III) crystallise in the triclinic space group (P-1) with one molecule in the asymmetric unit; such an observation is relatively rare, wherein all the polymorphic forms consist of *Z*′ = 1.

#### 3.1.2. Comparison of Crystal Structure of High-Pressure Form II with Form I and Form III

**Figure 2:**
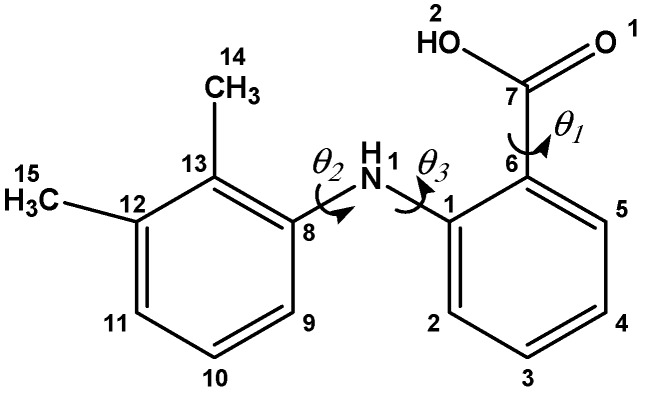
Mefenamic acid molecule, with the numbering scheme used in this work. Definition of torsional angles: *θ*_1_ is the angle involving O1-C7-C6-C1, *θ*_2_ is the angle involving C1-N1-C8-C13 and *θ*_3_ is the angle involving C6-C1-N1-C8.

The molecular structure of MA with the numbering scheme used in this work is shown in Figure 2. The conformation of the molecule in the crystal structure can be described by three torsional angles: *θ*_1_ (O1-C7-C6-C1), i.e. twisting of the carboxylic group with respect to the C6-C7 axis; *θ*_2_ (C1-N1-C8-C13), i.e. twisting of the second phenyl group with respect to the N1-C8 axis; and *θ*_3_ (C6-C1-N1-C8), i.e. twisting of bridging group relative to the C1-N2 axis. These torsional angles are shown by arrows in Figure 2 and are calculated by using the program MERCURY [32].

**Figure 3:**
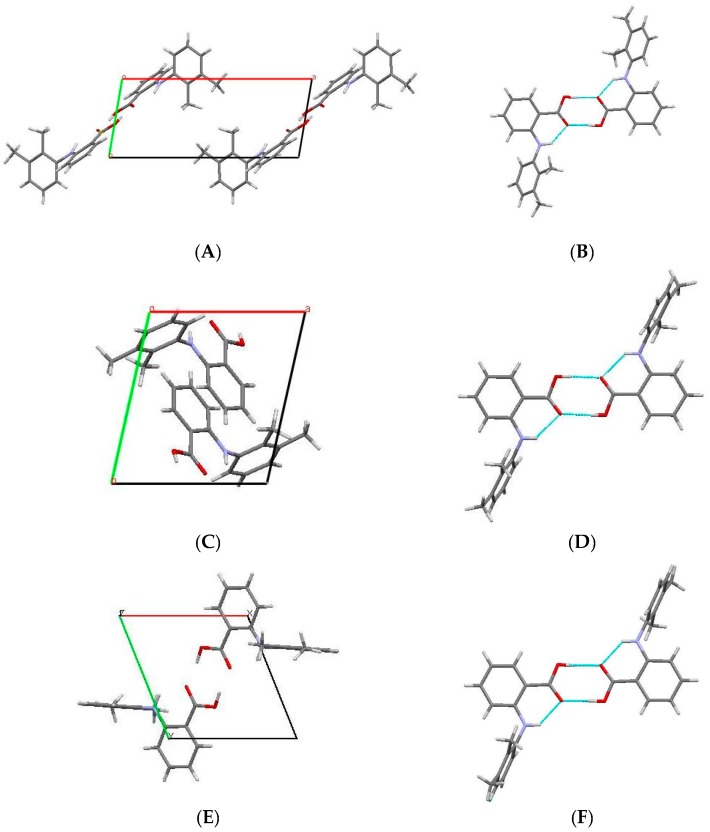
(**A**) Crystal packing diagram of Form I viewed down the *c*-axis; (**B**) Dimer unit of MA molecule in Form I; (**C**) Crystal packing diagram of high-pressure Form II viewed down the *c*-axis; (**D**) dimer unit of MA molecule in Form II; (**E**) Crystal packing diagram of Form III viewed down the *c*-axis; (**F**) Dimer unit of MA molecule in Form III.

The crystal packing diagram of Form I, II (high-pressure form) and Form III are compared in Figure 3. Although the polymorphs exhibit significant variation in the unit cell dimensions, the MA molecules pack as dimer units through an intermolecular hydrogen bond involving the carboxylic acid group (O2–H…O1) with an O2…O1. The length of this hydrogen bond is comparable in all the three forms with the value of ~1.68 Å. The phenyl ring bearing the carboxyl group is coplanar with the carboxyl- and bridging amino-groups. This coplanar conformation is stabilised by the resonance interaction and internal hydrogen bond between the amino- and carboxyl-groups [33]. The distance between the atoms (N1…O1) involved in this interaction in Form I, high-pressure Form II and III are 1.88, 2.01 and 2.09 Å, respectively.

The description of the hydrogen-bond pattern in all three forms according to the graph-set notation at the first level graph set is *N*_1_ = *S*(6)*R*_2_^2^ (8) (The graph-set notations are assigned by the method described by Bernstein et al. [34], and is obtained from PLUTO [35] and MERCURY [32]), where *S* and *R* motifs represents internal and external hydrogen bonds, respectively.

**Figure 4:**
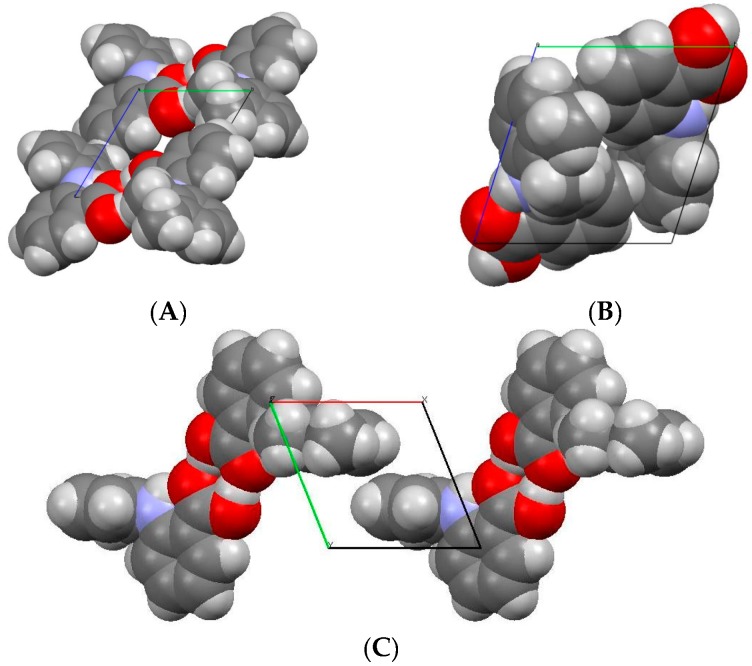
Space-filling diagrams for (**A**) Form I; (**B**) Form II; (**C**) Form III.

The comparison of space-filling diagrams (Figure 4) of the three forms indicates a more efficient packing of MA molecules in high-pressure Form II, as expected. The difference in the crystal packing arises from the torsional angle *θ*_2_ (Table 3), where the values changed significantly from −119.99° to −85.19°, whilst the deviation in the other two torsional angles, (*θ*_1_ and *θ*_3_) were very small. Due to changes in θ_2_, the carboxylic acid group and the amino group were no longer coplanar and as a result the length of the intramolecular hydrogen bond (N1...O1) increased from 1.82 to 2.01 Å.

#### 3.1.3. Decompression Studies

In order to find out the recovery of high-pressure Form II at ambient conditions, pressure was gradually decreased from 0.3 GPa at 293 K to ambient pressure, and no colour change or destruction of the crystal was observed. This crystal was then removed from gasket, placed on the goniometer and was successfully indexed as Form II, confirming its recovery to ambient condition. However, owing to poor quality and the damage done during unloading from the gasket, it was not possible to obtain sufficiently good data for full structure refinements. The crystal was also used as a seed in order to attempt the growth of single crystals of Form II at ambient pressure, but this resulted in the production of stable Form I instead. This suggests that presence of Form I seeds in solution may dominate the crystallisation process.

In order to demonstrate reproducibility, we found that repeated crystallisation at high pressure always resulted in Form II. These results are of great significance as they demonstrate that Form II can be prepared easily at high pressure and recovered successfully to ambient pressure. Furthermore, unlike in the study by Lee et al. [19], no additives were required. This may suggest that the Form II is the more thermodynamically stable Form at high pressure, mirroring the behaviour of paracetamol [36].

#### 3.1.4. IR Studies

**Figure 5:**
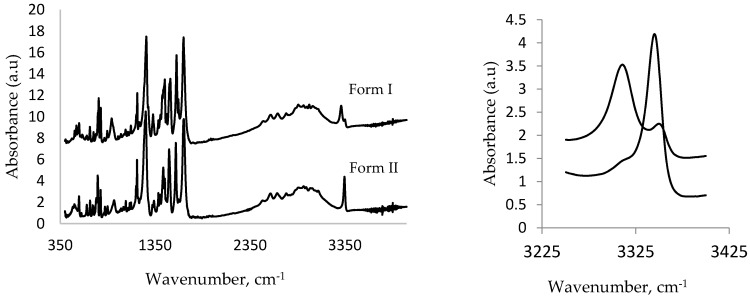
Comparison of IR absorption spectra (400–3500 cm^−1^) of MA Form I and high-pressure Form II (full spectra **left**), zoomed (**right**).

Figure 5 shows the comparison between the IR spectra of Form I and high-pressure Form II. An important spectral feature that can be used to distinguish between these polymorphs is the N–H stretching band (that occurs in the range of 3300–3350 cm^−1^) of the amino group that is associated with an intramolecular hydrogen bond with the carboxyl group (see Figure 3). On conversion to Form II, this band was shifted from 3312 cm^−1^ to a higher frequency at 3353 cm^−1^. This band is also significantly less broad than the initial band at 3312 cm^−1^. Both of these observations are consistent with the crystallographic results. In the case of Form I, the N–H bond is involved in a relative strong interaction with the carbonyl group (N...O distance is 2.63 Å). In Form II, this distance increases to 2.67 Å, indicating a weaker interaction caused by conformational changes. This results in the N–H bond becoming stronger and hence shifting to higher energy. These spectral changes are consistent with the quantum mechanical calculations conducted on the structure of MA [33].

## 4. Discussion

Mefenamic acid (MA) is an example of a polymorphic system where the single crystal of the stable form (Form I) can be obtained with relative ease, and its crystal structure has been known since 1976. However, single crystals of metastable forms (Forms II and III) are very difficult to obtain, and this is why their structure has been reported only recently [15]. Our attention was drawn to this compound by the observation that Form II is slightly denser than Form I and has better packing of molecules within the unit cell. MA molecules, having torsional flexibility (Figure 2), is a good candidate for such type of studies, where the rotation of one phenyl ring to other (torsional angle θ_2_) may result in polymorphic transition. Hence, our aim was to explore the effect of pressure on the polymorphic transformation of MA and also to explore whether the single crystal of Form II could be obtained reproducibly by high-pressure techniques.

At present, there are two known methods to obtain the metastable Form II; (i) the heating of Form I above the transition temperature; and (ii) the cooling of a supersaturated solution in *N*,*N*-Dimethylformamide or chloroform. The thermal method resulted in the formation of a polycrystalline mass, and the solution method resulted in conversion to Form I on cooling. A recently reported method involves the crystallisation of MA in the presence of an additive, flufenamic acid (FFA) [19]. Being structurally similar to Form I of MA, FFA blocked the nucleation process of Form I and resulted in production of Form II. This allowed the crystal structure of Form II to be determined for the first time. This procedure is tedious, however, and involves a number of controlled and lengthy processes (taking more than 20 days) and often results in failure. In an attempt to obtain a reliable single crystal of Form II, SeethaLekshmi and Guru Row performed a large number of crystallisation experiments by adopting different conditions and employing numerous solvents or solvent mixtures [15]. However, owing to the low solubility in the majority of solvents and solvent mixtures, their crystallisation experiments always resulted in Form I. Conversely, the only successful method was by the slow evaporation of a chloroform solution in a less humid condition, which yielded single crystals of Form II. This shows the elusive nature of Form II, which emphasises a need to explore alternate crystallisation conditions.

Our initial attempts to obtain Form II by recrystallisation from a solution, using a number of solvents (including chloroform) at ambient pressure conditions always resulted in the stable Form I (see Supplementary Materials). Different levels of supersaturation were also applied, but the results were the same; stable Form I was produced every time. Optical observation of the recrystallisation process showed that on crystallisation the solution turned yellowish-green first before the white precipitate appeared, which is an indication of the crystallisation of Form II. This may be explained by the fact that the initially nucleated metastable polymorph (Form II) dissolved rapidly into the surrounding phase and again nucleated as the stable Form I. All of these observations suggested that MA is an example of concomitant polymorphism, meaning two or more crystal forms that nucleate and grow simultaneously under identical conditions but end with the conversion of the metastable form to the most stable form [37]. Occasionally, in the case of concomitant polymorphs, the metastable form is not detected during the conventional screening process, as it remains in contact with the solution and may convert to the more stable form via a solution-mediated transformation process by means of dissolution and recrystallisation [38,39]. There was a need to explore alternative crystallisation conditions e.g., with the use of pressure as additional variable in conjunction with conventional strategies.

The compression behaviour of MA has been studied in connection with mechanical and dissolution properties [40], but very little attention has been paid to polymorphic transition under the conditions of high pressure. Yet, previous high-pressure studies have shown that hydrostatic compression can lead to a polymorphic transition, especially for those molecules with structural flexibility. Therefore, our first attempt was to compress the single crystal of the most stable form (i.e., Form I) in the presence of methanol-ethanol (4:1) mixture as a pressure-transmitting medium. Results showed a ~14% decrease in unit cell volume without any phase transition up to 2.50 GPa. A further increase in pressure to 3.0 GPa resulted in the disintegration of the single crystal, suggesting a destructive phase transition. This was confirmed by the recrystallisation from the solution under a high-pressure experiment, where Form II (denoted as high-pressure Form II) was generated at 0.3 GPa pressure. Further, a high quality single crystal of Form II was produced by this procedure, which was previously very difficult to achieve.

A comparison of the crystal structure of the high-pressure form with other forms showed that pressure encouraged the efficient packing of MA molecules and resulted in a denser structure. This was enabled by a change in the conformation of the MA molecule, where the torsional angle θ_2_ decreased from –119.99° to –85.18° in Form I and II, respectively. This conformational change also resulted in the weakening of the internal hydrogen bond as the length of this contact increased from 1.88 to 2.01 Å. This was also confirmed by the IR spectra of the two forms (Figure 5) where the N–H stretching frequency changed from 3312 to 3353 cm^−1^ as a result of the transformation from Form I to Form II.

## 5. Conclusions

A single crystal of Form II was obtained by the high-pressure crystallisation technique at 0.3 GPa. The example of mefenamic acid demonstrated that pressure can be used to induce polymorphism in organic compounds, where conformational flexibility in the structure may result in the production of polymorphs. The direct compression of a solid substance may encounter a kinetic barrier, which can be overcome by this process.

## Figures and Tables

**Table 1:** Lattice parameters for Form I with increasing pressure up to 2.50 GPa.

Pressure	Ambient	0.18 GPa	0.49 Gpa	0.90 GPa	1.67 GPa	2.50 GPa
Crystal system	Triclinic	Triclinic	Triclinic	Triclinic	Triclinic	Triclinic
Space group	*P*1̅	*P*1̅	*P*1̅	*P*1̅	*P*1̅	*P*1̅
*a* (Å)	6.7582(14)	6.775(2)	6.7120(13)	6.6432(3)	6.5642(13)	6.5153(13)
*b* (Å)	7.3391(15)	7.288(2)	7.2369(14)	7.1516(6)	7.1406(14)	7.0188(14)
*c* (Å)	14.3127(29)	14.303(15)	14.0762(28)	13.7098(8)	13.4047(27)	13.1342(26)
*α* (°)	76.69(3)	76.72(5)	77.02(3)	77.724(6)	77.888(3)	78.38(26)
*β* (°)	79.83(3)	79.11(5)	79.43(3)	78.962(4)	77.426(3)	77.11(3)
*γ* (°)	65.70(3)	65.55(2)	65.72(3)	65.606(6)	63.846(3)	62.82(3)
*V* (Å^3^)	626.94(31)	622.1(7)	604.1(31)	575.69(6)	545.78(2)	535.0(17)
Z	2	2	2	2	2	2
*T* (K)	298	298	298	298	298	298

**Table 2:** Crystallographic information of various polymorphs of mefenamic acid (MA) compared with high-pressure structure.

Parameter	MA II (high-pressure form) ^a^	MA II (CSD:XYANAC04) ^b^	MA III (CSD:XYANAC03) ^b^	MA I (CSD:XYANAC) ^c^
Chemical formula	C_15_H_15_NO_2_	C_15_H_15_NO_2_	C_15_H_15_NO_2_	C_15_H_15_NO_2_
Formula weight	241.29	241.29	241.29	241.29
Crystal system	*P*1̅	*P*1̅	*P*1̅	*P*1̅
Space group	Triclinic	Triclinic	Triclinic	Triclinic
*a* (Å)	7.7900(15)	7.7584(5)	7.723(2)	14.556
*b* (Å)	9.1890(18)	9.2772(6)	7.9340(10)	6.811
*c* (Å)	9.4120(19)	9.3991(4)	11.2320(10)	7.657
*α* (°)	106.751(10)	106.308(5)	83.590(10)	119.57
*β* (°)	92.287(12)	91.847(4)	80.940(10)	103.93
*γ* (°)	101.377(11)	101.856(5)	67.510(10)	91.30
*V* (Å^3^)	629.1(2)	632.52(6)	626.96)	631.766
*Z*	2	2	2	2
*T* (K)	298(2)	298(2)	298(2)	298
*R* (F_o_)	0.095	0.089	0.042	0.045
*R*_w_ (*F^2^_o_*)	0.095	0.302	0.109	-
Programme used	CRYSTALS	SHELX97	SHELX97	MULTAN

^a^ this study; ^b^ S. S. Lekshmi et al. [15]; ^c^ McConnell et al. [18], the lattice parameters are taken from McConnell et al. and are a different setting to our data.

**Table 3:** Comparison of torsional angles between Forms I and II of MA.

Torsional Angle	MA I [18]	MA II (This Study)	MA II [15]		MA III [15]
			*a* ^a^	*b* ^b^	
*θ*_1_	178.60	177.45	−177.43	−177.43	−177.38
*θ*_2_	−119.99	−85.18	−68.20	−71.01	−80.82
*θ*_3_	−179.34	−171.50	−176.32	−168.41	−179.55

MA molecule of Form II (produced by SeethaLekshmi and Guru Row [15]) shows structural disorder with ^a^ 55% occupancy; ^b^ 45% occupancy.

## Supplementary Materials

The following are available online at www.mdpi.com/1999-4923/9/2/16/s1, Table S1: Crystal, collection and refinement details for MA Form II at high pressure.
