# Supplementary material for: Accessing Mefenamic Acid Form II through High-Pressure Recrystallisation
Source: Pharmaceutics. 2017 May 16;9(2):16. doi: 10.3390/pharmaceutics9020016 (PMC5489933; doi:10.3390/pharmaceutics9020016)
Supplement: Supplementary file 1 [file pharmaceutics-09-00016-s001.pdf]

Supplementary Materials: Accessing Mefenamic Acid Form II through High-Pressure Recrystallisation

Nasir Abbas, Iain D. H. Oswald and Colin R. Pulham

Crystallographic Information Files for the structures: Mefenamic acid form II by high pressure.

Table S1. Crystal, collection and refinement details for MA Form II at high pressure.

Form II	
Crystal data	
Chemical formula	$C_{15}H_{15}NO_2$
M_r	241.29
Cell setting, space group	Triclinic, *P*-1
a, *b*, *c* (Å)	7.7900(15), 9.1890(18), 9.4120(19)
α, *β*, *γ* (°)	106.751(10), 92.287(12), 101.337(11)
V(Å^3)	629.1(2)
Z	2
D_x(Mg m^{-3})	1.27
Radiation type	Mo-*Kα*
No. of reflections for cell parameters	418
θ range (°)	2.272-20.870
μ (mm^{-1})	0.085
Temperature (K)	298(2)
Pressure (GPa)	0.3
Crystal form, colour	Block, colourless
Crystal size (mm)	0.1 × 0.1 ×0.15
Data collection	
Diffractometer	Bruker *SMART*
Data collection method	*ω* scans
Absorption correction	Multi-scan (SADABS)
T_{min}	0.630
T_{max}	1.000
No. of measured, independent and observed reflection	4003, 1210
Criterion for observed reflection	$I > 2\sigma(I)$
R_{int}	0.0269
θ_{max} (°)	20.87
Range of *h*, *k*, *l*	$-7 \rightarrow h \rightarrow 7$<br>$-9 \rightarrow k \rightarrow 9$<br>$-9 \rightarrow l \rightarrow 9$
Refinement	
Refinement on	*F*
R factor, *wR* factor, *S*	0.0953, 0.0942, 1.23
No. of reflection	418
No. of parameters	73
H-atom treatment	Riding
Weighting scheme	$w' \times [1 - (\Delta F_{obs} / 6 \times \Delta F_{est})^2]^2$<br>$w' = [P_0T_0'(x) + P_1T_1'(x) + ...P_{n-1}T_{n-1}'(x)]^{-1}$,<br>where P_i are the coefficients of a Chebychev series in $t_i(x)$,<br>and $x = F_{calc}/F_{calcmax}$.<br>$P_0 - P_{n-1} = 3.28 - 0.337 \times 1.88$
$(\Delta/\sigma)_{max}$	< 0.0001
$\Delta\rho_{max}$, $\Delta\rho_{min}$ (eÅ^{-3})	0.50, −0.46
Extinction method	none
